# The changes of gut microbiota after acute myocardial infarction in rats

**DOI:** 10.1371/journal.pone.0180717

**Published:** 2017-07-07

**Authors:** Ze-Xuan Wu, Su-Fang Li, Hong Chen, Jun-Xian Song, Yuan-Feng Gao, Feng Zhang, Cheng-Fu Cao

**Affiliations:** 1 Department of Cardiology, Peking University People’s Hospital, Beijing, China; 2 Beijing Key Laboratory of Early Prediction and Intervention of Acute Myocardial Infarction, Peking University People’s Hospital, Beijing, China; 3 Center for Cardiovascular Translational Research, Peking University People’s Hospital, Beijing, China; 4 Department of Cardiology, The First Affiliated Hospital of Sun Yat-Sen University, Guangzhou, China; South Texas Veterans Health Care System, UNITED STATES

## Abstract

Recent studies suggested that gut microbiota was involved in the development of coronary artery disease. However, the changes of gut microbiota following acute myocardial infarction (AMI) remain unknown. In this study, a total of 66 male Wistar rats were randomly divided into control, AMI and SHAM groups. The controls (n = 6) were sacrificed after anesthesia. The AMI model was built by ligation of left anterior descending coronary artery. The rats of AMI and SHAM groups were sacrificed at 12 h, 1 d, 3 d, 7 d and 14 d post-operation respectively. Gut microbiota was analyzed by 16S rDNA high throughput sequencing. The gut barrier injuries were evaluated through histopathology, transmission electron microscope and immunohistochemical staining. The richness of gut microbiota was significantly higher in AMI group than SHAM group at 7 d after AMI (P<0.05). Principal coordinate analysis with unweighted UniFrac distances revealed microbial differences between AMI and SHAM groups at 7 d. The gut barrier impairment was also the most significant at 7 d post-AMI. We further identified the differences of microorganisms between AMI and SHAM group at 7 d. The abundance of *Synergistetes* phylum, *Spirochaetes* phylum, *Lachnospiraceae* family, *Syntrophomonadaceae* family and *Tissierella Soehngenia* genus was higher in AMI group compared with SHAM group at 7 d post-operation (q<0.05). Our study showed the changes of gut microbiota at day 7 post AMI which was paralleled with intestinal barrier impairment. We also identified the microbial organisms that contribute most.

## Introduction

The role of gut microbiota in regulation of health and disease has been attracting increasing attention recently. The human gastrointestinal tract is estimated to contain approximately 100 trillion bacterial cells, belonging to 1,000 bacterial species.[1] The genome of all these microorganisms contains 100 times more genes than the human genome.[2] Recent studies showed that gut microbiota is involved in the development of coronary artery disease (CAD) through several mechanisms. Firstly, increase absorption of energy from gut may contribute to obesity and metabolic disturbances which in turn contribute to cardiovascular risk. This effect is partly mediated by short chain fatty acids (SCFAs), in particular butyrate, which are end-products of microbial fermentation of dietary fibers and play an important role in the energy harvest from the gut and maintaining the integrity of the gut barrier.[3, 4] The other mechanism involves the production of the proatherosclerotic metabolite, trimethylamine-*N*-oxide (TMAO). The metabolism of dietary phosphatidylcholine and L-carnitine by intestinal microbiota, results in the formation of the metabolite trimethylamine and further conversion to TMAO. TMAO was associated with atherosclerosis and elevated plasma levels of TMAO predict an increased risk of cardiovascular disease including incidence myocardial infarction.[5–8]

Although stable over long periods, the compositions and functions of the gut microbiota are influenced by many factors including genetics, age, diet, stress, probiotics/ prebiotics, antibiotics and health status.[9–13] AMI, caused by the necrosis of myocardium due to prolonged ischemia, was one of the most critical stress to the patients. Intestinal barrier dysfunction was common in AMI patients due to multiple factors including intestinal hypoperfusion, weakened regeneration ability of intestinal mucosa for long term bedridden and reduced food intake, as well as excessive alkalization and intestinal bacterial over-breeding caused by the use of proton pump inhibitor. [14, 15] The integrity of gut barrier is closely linked to gut microbiota with the tight junction being the gatekeepers of the intestinal mucosal barrier.[16, 17] Gut barrier impairment may lead to alteration of gut microflora, in turn, gut microbiota can influence the integrity of the intestinal epithelium as well as the gut barrier function.[18]

However, whether gut microbiota will change or not following AMI remains unclear. Previous studies reported that the intestinal predominant microbiota significantly altered in AMI patients, while animal models showed no significant shifts after 6 weeks of sustained coronary artery ligation.[19, 20] The conflict maybe results from the simplicity of one single time point and the inconsistency of observational time in different studies. Furthermore, the stress induced by the intervention may be a confounding factor. Therefore, our study aims to figure out the changes of gut microbiota in a rat model of AMI with the setting of SHAM groups.

In recent years, high-throughput 16S rDNA sequencing has greatly contributed to the research of gut microbiota.[21] Therefore, we used a rat model of AMI, built by sustained ligation of coronary artery, as well as high-throughput sequencing and HiSeq2500 PE250 platform to characterize the changes of both gut microbial communities and intestinal barrier. This study will provide insights and experimental basis for the treatment of AMI in the future.

## Materials and methods

### Animals

Male Wistar rats weighing 230-280g (7 to 8 weeks old) were maintained in a temperature controlled room with a 12-hour light/ dark cycles. This study was carried out in strict accordance with the recommendations in the Guide for the Care and Use of Laboratory Animals of the National Institutes of Health. The protocol was approved by the Committee on the Ethics of Animal Experiments of Peking University People’s Hospital (Permit Number: 2015–29).

### Establishment of AMI model

Rats were randomly divided into three groups: control group (CON group), AMI group and SHAM group. The CON group (n = 6) were sacrificed just after over dose anesthesia. The AMI model was built by ligating the left anterior descending artery (LAD) as described previously.[22] Briefly, all surgery was performed under isoflurane inhalation anesthesia and mechanical ventilation. A left lateral thoracotomy was performed and a ligature using 6–0 prolene was placed around the LAD beneath the left atrium. AMI was deemed successful based on regional cyanosis of the myocardial surface distal to the suture, accompanied by elevation of the ST segment on ECG. The SHAM group underwent the same procedure except coronary artery ligation. Caprofen jelly was added the day before and after operation.

The AMI and SHAM group were further divided into 5 subgroups respectively, which were euthanized at 12 h, 1 d, 3 d, 7 d and 14 d after operation. Every subgroup included 6 rats. Feces sample and tissue sample were harvested for further analysis. Rats were then killed by over dose anesthesia of isoflurane inhalation.

### Fecal sampling, sequencing and microbial analysis

Fecal samples from the rectum were collected in a sterile tube immediately after euthanasia and stored at -80°C until processing. DNA was extracted using CTAB/SDS method as described previously.[23] Hypervariable region V4 of 16S rRNA genes was amplified using forward primer 515F (GTGCCAGCMGCCGCGGTAA) and reverse primer 806R (GGACT CHVGGGTWTCTAAT). PCR amplicons were sequenced with Illumina HiSeq2500 platform. Raw sequence data were filtered, processed and analyzed according to the QIIME (V1.7.0) quality controlled process. [24, 25] Sequences with ≥97% similarity were assigned to the same operational taxonomic units (OTUs). Taxonomic annotation was made using RDP classifier (Version 2.2, http://sourceforge.net/projects/rdp-classifier/) algorithm and the GreenGene Database (http://greengenes.lbl.gov/cgi-bin/nph-index.cgi).[26, 27] 16S rRNA gene sequences were analyzed with the QIIME (Version1.7.0) software package and displayed with R software (Version 2.15.3). Alpha diversity is applied in analyzing complexity of species diversity for a sample through 3 richness estimators including observed species, ACE (abundance-based coverage estimator) (http://www.mothur.org/wiki/Ace) and Chao1 (http://www.mothur.org/wiki/Chao). Principal coordinates analyses (PCoA) plots using unweighted UniFrac were created through QIIME. Differentially significant features at each level were further identified using linear discriminant analysis (LDA) along with effect size measurements (LEfSe) with a cut-off LDA score (log10) of 4.0 or greater.

### Intestinal histopathologic analysis

The ileum 5 cm above the cecum was cut off immediately after the rats were killed and fixed with 4% formaldehyde. Paraffin-embedded samples were cut and stained using hematoxylin-eosin (H&E) stain to detect morphologic changes.

The Chiu pathologic scores of mucosal injuries were used to evaluate the degree of intestinal histological injury. The scores value from 0 to 5 where 0 normal mucosa; 1, development of sub epithelial (Gruenhagen’s) spaces; 2, extension of the sub epithelial space with moderate epithelial lifting from the lamina propria; 3, extensive epithelial lifting with occasional denuded villi tips; 4, denuded villi with exposed lamina propria and dilated capillaries; and 5, disintegration of the lamina propria, hemorrhage, and ulceration.[28]

### Transmission electron microscopy

The ileum samples also 5 cm above the cecum were cut into 1 mm × 1 mm × 1 mm sections, pre-fixed with 3% glutaraldehyde, fixed with 1% osmium tetroxide, dehydrated in acetone (50%, 70%, 90% and 100%) and then embedded in Epon 812. Semi-thin sections were used for optical positioning, whereas ultra-thin sections were used for double staining with uranyl acetate and lead citrate. The sections were observed by electron microscopy (FEI TECNAI SPIRIT).

### Immunohistochemical stain for tight junction protein

Paraffin-embedded rat ileum sections were used for immunohistochemical stain (IHC). The reaction was carried out for occludin (intracellular tight junction protein) visualization as previously described.[17] The primary rabbit polyclonal anti-occludin antibody was bought from Abcam (ab31721).

### Statistical analysis

Continuous nonparametric data were analyzed using a Mann–Whitney U, and the results are presented as the median and interquartile range (IQR). Continuous parametric data, presented as numbers and percentage or as the mean ± standard deviation, were analyzed with a student’s t test. Categorical data were analyzed using a Chi-square test. The difference of abundance among multiple groups were compared using metastats software. For each feature i, the p value between two groups were calculated using t-tests. To control the false discovery rate (FDR), the q value was corrected using Benjamini FDR correction. The formula is as follows: q-value (i) = p (i) *length (p) /rank (p).[29]Differences were considered statistically significant at p < 0.05 or q<0.05. GraphPad Prism statistical software version 5 was used for analysis.

## Results

### 16S rDNA profiling of gut microbiota in rats after AMI

We performed 16S rDNA sequencing on the fecal samples collected from CON group at baseline, and from AMI and SHAM groups at 12 h, 1 d, 3 d, 7 d and 14 d after operation. In total, we analyzed 66 samples and obtained 3,757,434 raw tags. After quality control, a total of 3,631,027 sequences were obtained and assigned into 2014 OTUs with 97% similarity or greater for this analysis.

**Fig 1:**
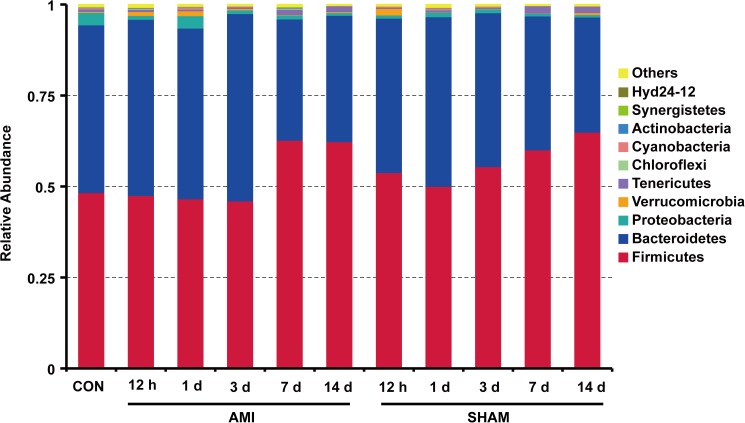
The relative abundance of gut microbiota at phylum level in different groups (n = 6 for each column). CON, control group; AMI, acute myocardial infarction group; SHAM, sham group.

The microbial compositions of different groups at phylum level showed that *Firmicutes* (54.38%) and *Bacteroidetes* (41.88%) were the two most dominant phyla, accounting for more than 96% of microorganisms (Fig 1). The relative abundance of *Firmicutes* was increased and *Bacteroidetes* reduced at 7 d, 14 d compared to 12 h, 1 d and 3 d within AMI groups (P<0.05). However, there was no significant difference in proportions of the two major phyla between AMI and SHAM groups at the same time point (P>0.05), suggesting no significant microbial changes at phylum level post-AMI.

### Changes of gut microbiota after AMI

**Fig 2:**
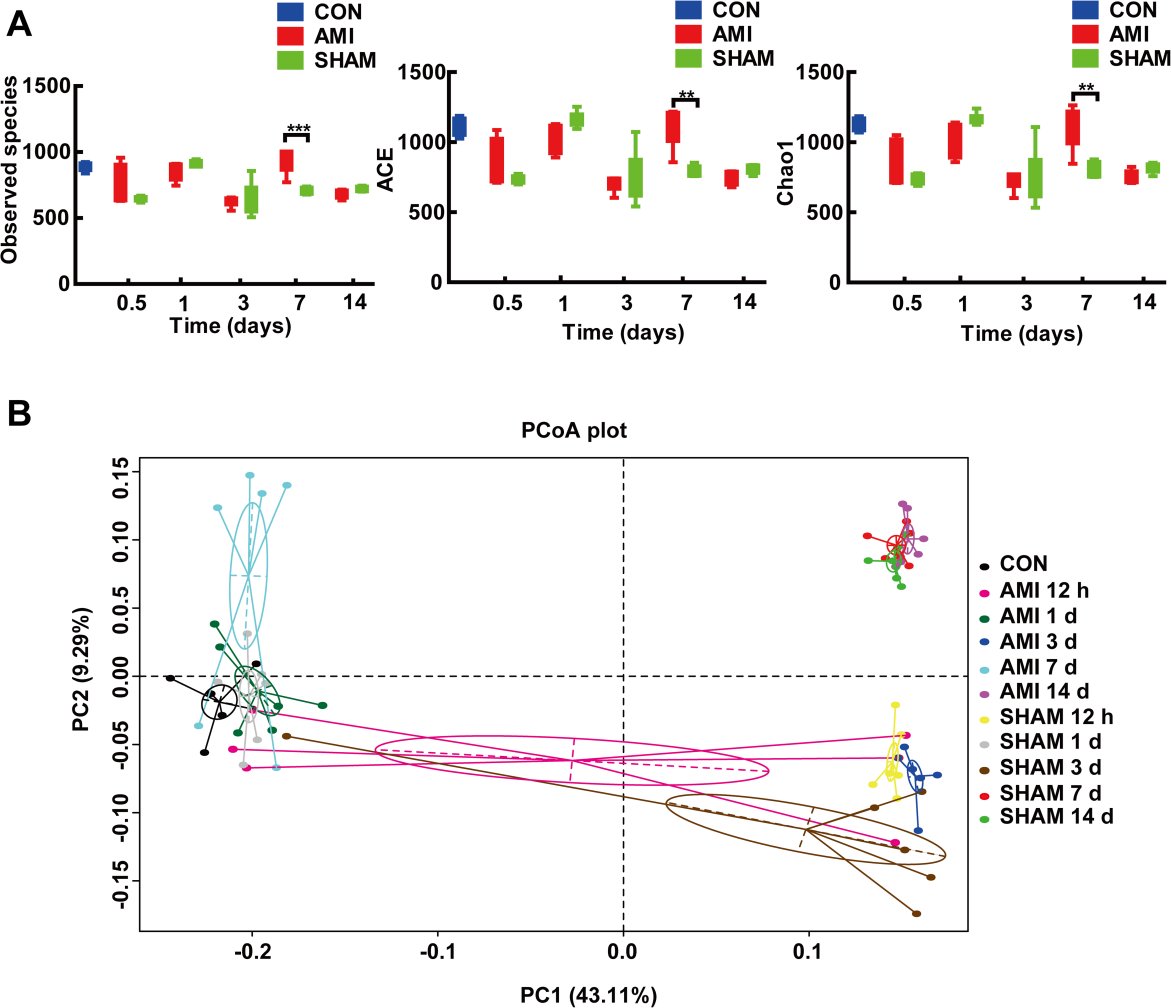
The microbial changes after AMI. **(**A) The richness index (including Observed species, ACE and Chao1) in different groups. The richness of AMI group was significantly higher than that of SHAM group at 7 d post-operation. ** P<0.01; *** P<0.001. (B) Principal coordinate analysis (PCoA) of gut microbiota showed that the microbial communities of AMI group at 7 d post-AMI clustered distinctly from SHAM group at 7 d. One circle represents microbiota composition (all phyla combined) in one subgroup (n = 6, one dot represents one sample). CON, control group; AMI, acute myocardial infarction group; SHAM, sham group.

To determine the effect of AMI on the richness of gut microbiota with ischemic time, we compared index including observed species, ACE and Chao1 at different time points. The richness of gut microbiota fluctuated over the ischemic period and showed a significant difference between AMI and SHAM group at 7 d post-operation (Fig 2A). The observed species (929.2± 87.8 vs. 709.3 ± 24.8, P<0.001), ACE (1091.4 ± 131.2 vs. 800.6 ± 36.7, P<0.001) and Chao1(1087.6 ± 145.0 vs. 813.0 ± 51.4, P = 0.001) of AMI group were significantly higher than those of SHAM group at 7 d. The observed species (709.3± 24.8 vs. 884.7 ± 30.4, P<0.001), ACE (800.6 ± 36.7 vs. 1106.6 ± 61.0, P<0.001) and Chao1 (813.0 ± 51.4 vs. 11167.0 ± 46.8, P<0.001) of SHAM group at 7 d were significantly lower than those of CON group. However, there was no significant difference between CON and AMI group at 7 d (P>0.05).

To further identify the time-point when microbial communities were most significantly changed after AMI, we performed principal coordinate analyses (PCoA) of the unweighted UniFrac distance matrics (Fig 2B). PC1 explained 43.11% and PC2 explained 9.29% of the variance. Rats at the same ischemic time are more likely to cluster together. Obvious microbial differences were confirmed between AMI and SHAM groups at 7 d when they clustered distinctly.

These results suggested that gut microbiota was markedly changed at 7 d post-AMI.

### Changes of microbial communities at 7 d after AMI

**Fig 3:**
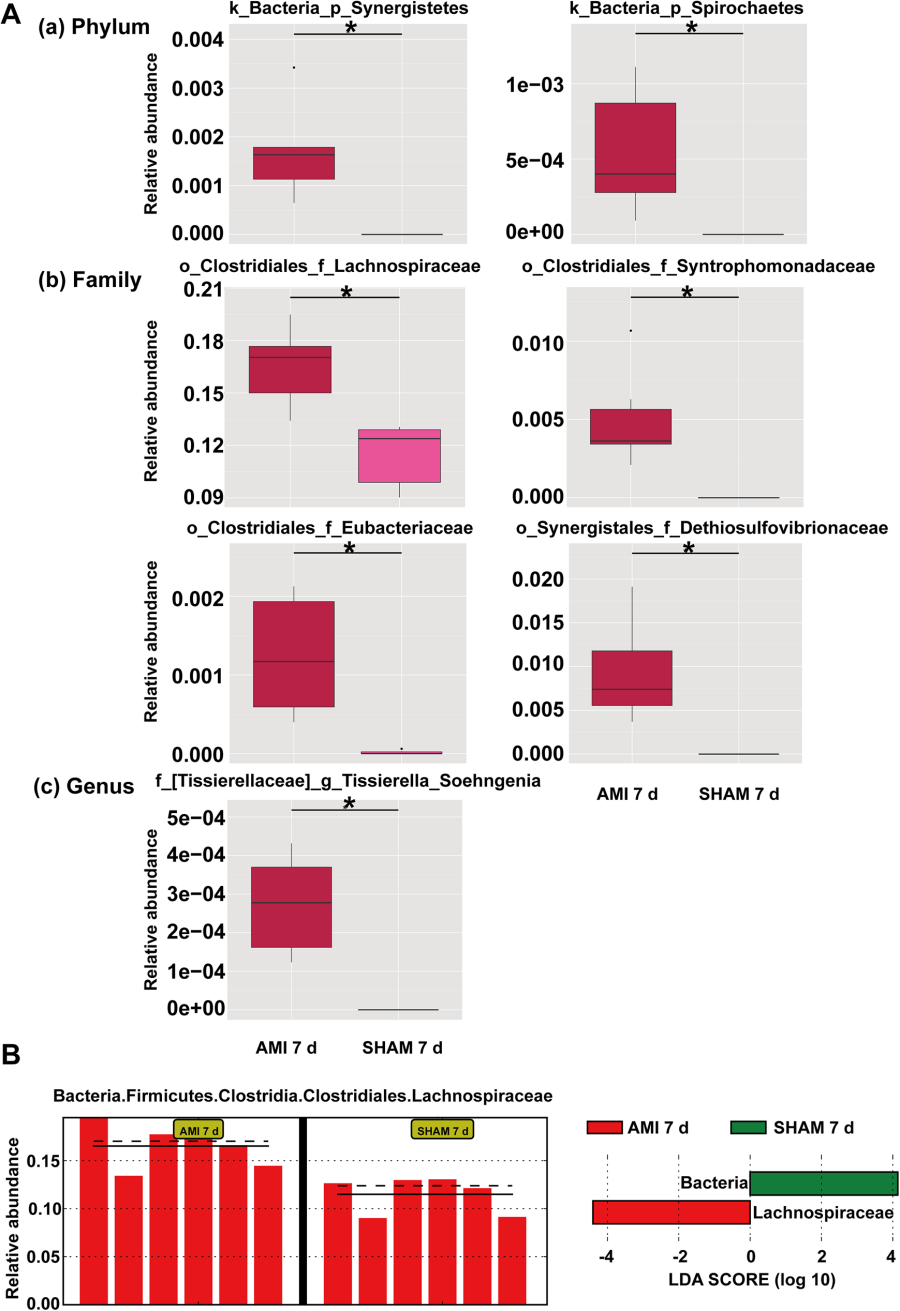
The changes of microbial communities at 7 d after AMI. (A) The microbial differences between AMI 7 d and SHAM 7 d group at phylum (a), family(b) and genus(c) level. The abundance difference between the two groups was showed by q value which was corrected by Benjamini discovery rate correction. * represents q<0.05, k kingdom, p phylum, o order, f family, g genus. (B) LEfSe analysis between AMI-7d and SHAM-7d group showed enrichment for *Lachnospiraceae* family in AMI group. CON, control group; AMI, acute myocardial infarction group; SHAM, sham group.

Since the microorganisms were significantly altered at 7 d after AMI as mentioned above, we then analyzed the microbial differences between AMI and SHAM group at 7 d to identify the potential microbial biomarker of AMI (Fig 3). We found that the relative abundances of *Synergistetes* (0.002 ±0.000 vs. 0, q = 0.019) and *Spirochaetes* (0.001 ± 0.000 vs. 0, q = 0.050) at phylum level, *Lachnospiraceae* (0.165 ± 0.009 vs. 0.115 ± 0.008, q = 0.027), *Syntrophomonadaceae* (0.005 ± 0.001 vs. 0, q = 0.027), *Eubacteriaceae* (0.001 ± 0.000 vs. 0, q = 0.027) and *Dethiosulfovibrionaceae* (0.001 ± 0.000 vs. 0, q = 0.027) at family level, and *Tissierella Soehngenia* (0.001 ± 0.000 vs. 0, q = 0.037) at genus level were significantly higher compared to SHAM group (Fig 3A).

Analysis with LEfSe also confirmed the enrichment for *Lachnospiraceae* family in AMI group at 7d compared with SHAM group (Fig 3B).

### Changes of gut barrier after AMI

**Fig 4:**
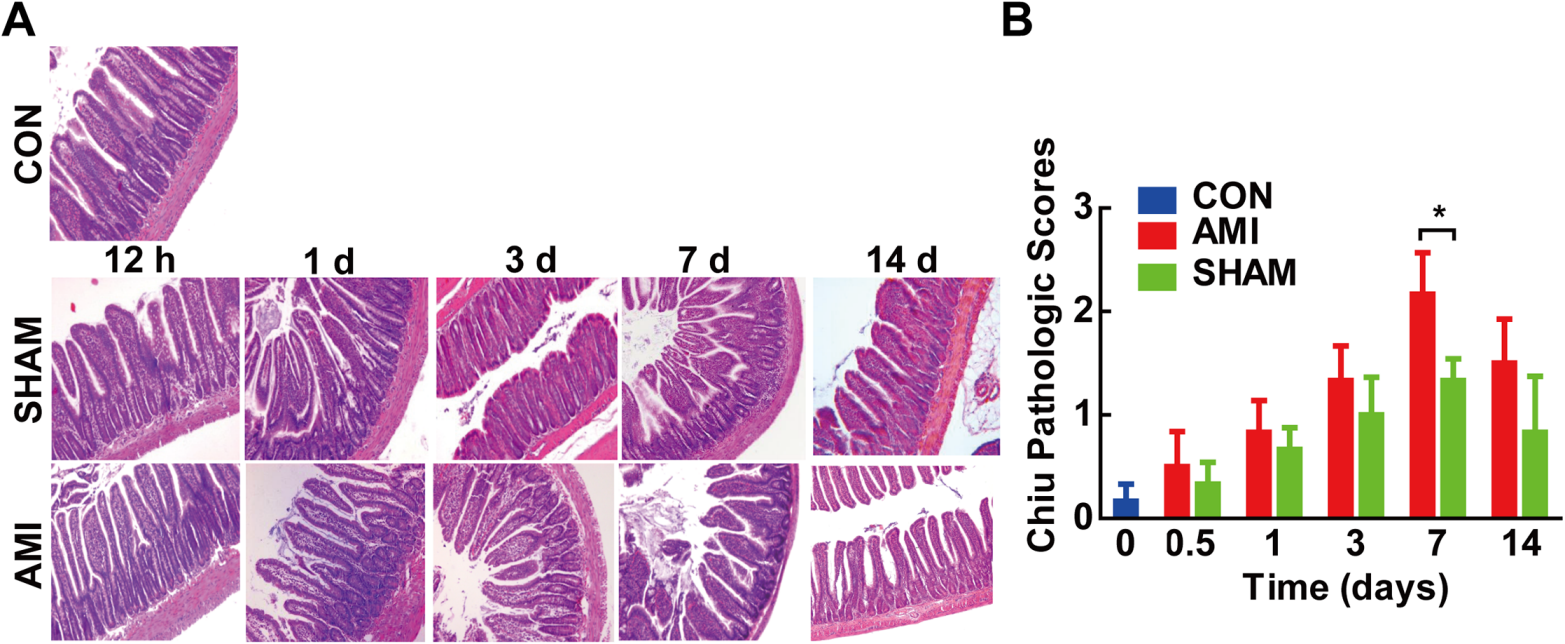
Changes of gut barrier in different groups. (A) Histopathological examination of rats’ distal ileum. (H&E stain×100) showed mucosal injury at 7 d post-AMI. (B) Chiu pathological scores revealed markedly higher scores in AMI 7 d group. CON, control group; AMI, acute myocardial infarction group; SHAM, sham group. *P<0.05.

Since the integrity of gut barrier is closely linked to alteration of gut microbiota, we further evaluate the changes of gut barrier after AMI (Fig 4).

Histological measurement of ileal morphology revealed significant effects of AMI on the intestinal particularly at 7 d post-AMI (Fig 4A). The microvilli became rough, dull, irregularly arranged and the epithelial cells of intestinal villus partly shed in AMI groups compared to SHAM groups. The Chiu pathological scores were significantly higher in AMI groups than those of SHAM groups at 7 d post-AMI (2.33± 0.82 vs. 1.00 ±0.89, P = 0.032) (Fig 4B).

Similar changes were observed in the ultrastructure (S1 Fig). The microvilli of intestinal epithelial cell became short, sparse, irregularly arranged at 7 d post-AMI and returned to normal after 2 weeks. Expression of occludin (tight junction protein) was also decreased at 7 d after AMI and gradually returned to normal 2 weeks post-AMI (S2 Fig).

These results showed significant gut barrier impairment at 7 d post-AMI, which was paralleled with the alteration of gut microbiota.

## Discussion

Previous studies have demonstrated that gut microbiota is linked to coronary artery disease. However, when and how the microbial communities alter post-AMI remains unclear. In this study, we observed the changes of gut microbiota and gut barrier post-AMI in a rat model. We found that the gut microbiota were significantly changed as early as 7 d post-AMI, paralleled with gut barrier impairment. The richness of gut microbiota was significantly higher in AMI group than SHAM group at 7 d after AMI. We also identified the microorganisms that significantly changed at 7 d after AMI.

There are many confounding factors influencing the changes of gut microbiota, such as surgical stress. As previous study showed, even handling pups for experimental purposes, without gavage, may induce enough stress to alter the murine gut microbiota. [30] In our study, the richness of SHAM group at day 7 was decreased compared with CON group (Fig 2), which may be explained by the invasive operation. Therefore, to confirm the true effect of AMI on gut microbiota, we used SHAM group instead of CON group for control. The procedures of sham group are as same as AMI group except the ligation of coronary artery. Therefore, other than the ligation, the potential confounding factors are controlled. Besides, caprofen jelly was added before and after operation to make sure the stress almost the same. We finally confirmed the effect of AMI on gut microbiota and found remarkably higher richness in AMI group than SHAM group at day 7 after AMI.

The mechanisms of gut microbiota alteration post-AMI remainunclear. Since the gut barrier impairment was paralleled with microbial alteration in our study, the integrity of gut barrier may play a role in the translocation of gut microbiota. Another possible mechanism is hemodynamic changes in the intestines post AMI. The gut is a blood-demanding organ, and villi (and microvilli) are prone to functional ischemia due to reduced blood flow.[31] In the setting of heart failure following AMI, substantial hemodynamic changes, such as hypoperfusion and congestion in the intestines, can alter gut morphology, leading to increase of gut permeability, intestinal dysfunction, and possibly translocation of gut microbiota.[32, 33] The bacterial metabolites translocated into the blood circulation will further promote systemic inflammatory response, increase the level of inflammatory mediators such as TNF-α, IL-1 and IL-6, and in turn aggravate the intestinal mucosal injury.[34]

The gut microbiota tends to change in multiple diseases characterized with increase of opportunistic bacteria and decrease of probiotic bacteria. [35, 36] In our study, we observed the increase of *Synergistetes* pylum and *Lachnospiraceae* family post-AMI. *Synergistetes*, one of the opportunistic bacteria, is involved in the pathogenesis of periodontitis and peri-implantitis.[1, 37] Most *Lachnospiraceae* are butyrate-producers. Butyrate, a short chain fatty acid, is the main energy supply for colonic epithelia cells. [38] However, whether these gastrointestinal nutrients can provide energy for the myocardial infarcted region remains unclear. Sodium butyrate (NaB, C_4_H_7_O_2_Na), a nutrient whose active component was butyric acid, was reported to ameliorate cardiac dysfunction in rats post AMI through directly injecting into the cardiac ischemic zones. The possible mechanisms include inhibiting the generation of reactive oxygen species, autophagy and angiogenesis promotion.[39] [40]The increase of *Lachnospiraceae* showed in our study may be a feedback mechanism for the lack of energy in the myocardium post AMI. Therefore, timely replenishment of nutrients like butyrate or the butyrate-producers such as *Lachnospiraceae* following AMI may be a potential therapeutic option for AMI treatment. However, further studies are warranted to investigate whether oral or intravenous supplementation is as equally effective as intramyocardial administration.

We also found the increase of other pathogenic bacteria including *Spirochaetes* phylum, *Syntrophomonadaceae* family, *Eubacteriaceae* family and *Tissierella Soehngenia* genus after AMI. Both syphilis and Lyme disease are caused by *Spirochaetes*. [40, 41] The genus *Tissierella Soehngenia* belongs to the phylum *Firmicutes*, the *Clostridiales* and the family *Tissierellaceae*, which was also reported to be associated with infection. [42] Both *Syntrophomonadaceae* and *Eubacteriaceae* family belong to *Clostridiales* order, *Firmucutes* phylum. However, the role of these bacteria played in acute myocardial infarction remain unknown and further studies are still needed to verify.

Our study provided the experimental evidence for the changes of intestinal permeability and gut microbiota. We also identified the microorganisms that may correlated with energy supply post AMI. However, there are still some shortcomings in this study. Firstly, the AMI model in our study was based on ligation of coronary artery, which was not entirely consistent with clinical atherosclerosis plaque rupture. Secondly, the compositions of intestinal flora in rats were quite different from that of humans, so the results cannot be fully generalized to the population. Thirdly, due to the strains that were not culturable and no commercial ways to buy, we don’t further verify the potential biological function of the OTUs that were significantly related to AMI in our study. And we didn’t analyze the changes of SCFAs including butyrate and lactate. Therefore, further study was still needed targeting the gut microbiota and related metabolites, to establish a causal and clinically relevant relationship between the gut microbiome and AMI.

## Conclusions

Our study showed the changes of gut microbiota at day 7 post AMI which was paralleled with intestinal barrier impairment. We also identified the microbial organisms that were significantly altered. Our study provides the experimental basis for the study on the relationship between the AMI and intestinal flora.

## Supporting information

S1 Fig Changes of gut barrier under transmission electron microscopy in different groups. Transmission electron microscopy of ultrastructures of ileum (bar = 2.5 μm) showed that microvilli became short, sparse, irregularly arranged at 7 d post-AMI (white arrow shows the tight junction). A. Control group; B, D, F, H, J represents SHAM group at 12 h, 1 d, 3 d, 7 d, 14 d respectively; C, E, G, I, K represents AMI group at 12 h, 1 d, 3 d, 7 d, 14 d respectively.
(TIF)

S2 Fig Expression of tight junction protein in different groups. Immunohistochemical stain of tight junction protein occludin showed reduced expression of occludin (red arrow) in AMI group at 7 d post AMI.
(TIF)

## References

[1] Marchandin H, Damay A, Roudiere L, Teyssier C, Zorgniotti I, Dechaud H, et al Phylogeny, diversity and host specialization in the phylum Synergistetes with emphasis on strains and clones of human origin. Research in microbiology. 2010;161(2):91–100. doi: 10.1016/j.resmic.2009.12.008 . 20079831 10.1016/j.resmic.2009.12.008

[2] Backhed F, Ley RE, Sonnenburg JL, Peterson DA, Gordon JI. Host-bacterial mutualism in the human intestine. Science. 2005;307(5717):1915–20. doi: 10.1126/science.1104816 . 15790844 10.1126/science.1104816

[3] Sonnenburg JL, Backhed F. Diet-microbiota interactions as moderators of human metabolism. Nature. 2016;535(7610):56–64. doi: 10.1038/nature18846 . 27383980 10.1038/nature18846 PMC5991619

[4] Troseid M. Gut microbiota and acute coronary syndromes: ready for use in the emergency room? Eur Heart J. 2017 doi: 10.1093/eurheartj/ehx005 . 28159962 10.1093/eurheartj/ehx005 PMC5381592

[5] Khan MS, Bawany FI, Khan A. Intestinal microbiota produced trimethylamine-N-oxide can increase the risk of cardiovascular disease. JPMA The Journal of the Pakistan Medical Association. 2014;64(4):488 . 24864657

[6] Koeth RA, Wang Z, Levison BS, Buffa JA, Org E, Sheehy BT, et al Intestinal microbiota metabolism of L-carnitine, a nutrient in red meat, promotes atherosclerosis. Nature medicine. 2013;19(5):576–85. doi: 10.1038/nm.3145 ; PubMed Central PMCID: PMC3650111. 23563705 10.1038/nm.3145 PMC3650111

[7] Ussher JR, Lopaschuk GD, Arduini A. Gut microbiota metabolism of L-carnitine and cardiovascular risk. Atherosclerosis. 2013;231(2):456–61. doi: 10.1016/j.atherosclerosis.2013.10.013 . 24267266 10.1016/j.atherosclerosis.2013.10.013

[8] Troseid M, Ueland T, Hov JR, Svardal A, Gregersen I, Dahl CP, et al Microbiota-dependent metabolite trimethylamine-N-oxide is associated with disease severity and survival of patients with chronic heart failure. J Intern Med. 2015;277(6):717–26. doi: 10.1111/joim.12328 . 25382824 10.1111/joim.12328

[9] Yatsunenko T, Rey FE, Manary MJ, Trehan I, Dominguez-Bello MG, Contreras M, et al Human gut microbiome viewed across age and geography. Nature. 2012;486(7402):222–7. doi: 10.1038/nature11053 ; PubMed Central PMCID: PMC3376388. 22699611 10.1038/nature11053 PMC3376388

[10] Voreades N, Kozil A, Weir TL. Diet and the development of the human intestinal microbiome. Frontiers in microbiology. 2014;5:494 doi: 10.3389/fmicb.2014.00494 ; PubMed Central PMCID: PMC4170138. 25295033 10.3389/fmicb.2014.00494 PMC4170138

[11] Jernberg C, Lofmark S, Edlund C, Jansson JK. Long-term impacts of antibiotic exposure on the human intestinal microbiota. Microbiology. 2010;156(Pt 11):3216–23. doi: 10.1099/mic.0.040618-0 . 20705661 10.1099/mic.0.040618-0

[12] Dinan TG, Cryan JF. Regulation of the stress response by the gut microbiota: implications for psychoneuroendocrinology. Psychoneuroendocrinology. 2012;37(9):1369–78. doi: 10.1016/j.psyneuen.2012.03.007 . 22483040 10.1016/j.psyneuen.2012.03.007

[13] Bull MJ, Plummer NT. Part 1: The Human Gut Microbiome in Health and Disease. Integrative medicine. 2014;13(6):17–22. ; PubMed Central PMCID: PMC4566439. 26770121 PMC4566439

[14] Freis ED, Schnaper HW, Johnson RL, Schreiner GE. Hemodynamic alterations in acute myocardial infarction. I. Cardiac output, mean arterial pressure, total peripheral resistance, central and total blood volumes, venous pressure and average circulation time. The Journal of clinical investigation. 1952;31(2):131–40. doi: 10.1172/JCI102584 ; PubMed Central PMCID: PMC436393. 14907892 10.1172/JCI102584 PMC436393

[15] Mao Y, Wang SQ, Mao XB, Zeng QT, Li YS. Intestinal barrier function in patients with acute myocardial infarction and the therapeutic effect of glutamine. Int J Cardiol. 2011;146(3):432–3. doi: 10.1016/j.ijcard.2010.10.102 . 21094546 10.1016/j.ijcard.2010.10.102

[16] Bischoff SC, Barbara G, Buurman W, Ockhuizen T, Schulzke JD, Serino M, et al Intestinal permeability—a new target for disease prevention and therapy. BMC Gastroenterol. 2014;14:189 doi: 10.1186/s12876-014-0189-7 ; PubMed Central PMCID: PMCPMC4253991. 25407511 10.1186/s12876-014-0189-7 PMC4253991

[17] Kosik-Bogacka DI, Kolasa A, Baranowska-Bosiacka I, Marchlewicz M. Hymenolepis diminuta: the effects of infection on transepithelial ion transport and tight junctions in rat intestines. Experimental parasitology. 2011;127(2):398–404. doi: 10.1016/j.exppara.2010.09.001 . 20850436 10.1016/j.exppara.2010.09.001

[18] Tran CD, Grice DM, Wade B, Kerr CA, Bauer DC, Li D, et al Gut permeability, its interaction with gut microflora and effects on metabolic health are mediated by the lymphatics system, liver and bile acid. Future microbiology. 2015;10(8):1339–53. doi: 10.2217/FMB.15.54 . 26234760 10.2217/FMB.15.54

[19] Huang Y, Tan C. Analysis of the intestinal predominant microbiota in acute myocardial infarction patients. Chinese Journal of Microecology. 2014;26(9):1004–8. doi: 10.13381/j.cnki.cjm.201409004

[20] Gan XT, Ettinger G, Huang CX, Burton JP, Haist JV, Rajapurohitam V, et al Probiotic administration attenuates myocardial hypertrophy and heart failure after myocardial infarction in the rat. Circulation Heart failure. 2014;7(3):491–9. doi: 10.1161/CIRCHEARTFAILURE.113.000978 . 24625365 10.1161/CIRCHEARTFAILURE.113.000978

[21] Caporaso JG, Lauber CL, Walters WA, Berg-Lyons D, Lozupone CA, Turnbaugh PJ, et al Global patterns of 16S rRNA diversity at a depth of millions of sequences per sample. Proceedings of the National Academy of Sciences of the United States of America. 2011;108 Suppl 1:4516–22. doi: 10.1073/pnas.1000080107 ; PubMed Central PMCID: PMC3063599. 20534432 10.1073/pnas.1000080107 PMC3063599

[22] Yang B, Lin H, Xiao J, Lu Y, Luo X, Li B, et al The muscle-specific microRNA miR-1 regulates cardiac arrhythmogenic potential by targeting GJA1 and KCNJ2. Nature medicine. 2007;13(4):486–91. doi: 10.1038/nm1569 . 17401374 10.1038/nm1569

[23] Smith GL, Sansone C, Socransky SS. Comparison of two methods for the small-scale extraction of DNA from subgingival microorganisms. Oral microbiology and immunology. 1989;4(3):135–40. Epub 1989/09/01. . 2639297 10.1111/j.1399-302x.1989.tb00240.x

[24] Bokulich NA, Subramanian S, Faith JJ, Gevers D, Gordon JI, Knight R, et al Quality-filtering vastly improves diversity estimates from Illumina amplicon sequencing. Nature methods. 2013;10(1):57–9. doi: 10.1038/nmeth.2276 ; PubMed Central PMCID: PMC3531572. 23202435 10.1038/nmeth.2276 PMC3531572

[25] Caporaso JG, Kuczynski J, Stombaugh J, Bittinger K, Bushman FD, Costello EK, et al QIIME allows analysis of high-throughput community sequencing data. Nature methods. 2010;7(5):335–6. doi: 10.1038/nmeth.f.303 ; PubMed Central PMCID: PMC3156573. 20383131 10.1038/nmeth.f.303 PMC3156573

[26] DeSantis TZ, Hugenholtz P, Larsen N, Rojas M, Brodie EL, Keller K, et al Greengenes, a chimera-checked 16S rRNA gene database and workbench compatible with ARB. Applied and environmental microbiology. 2006;72(7):5069–72. doi: 10.1128/AEM.03006-05 ; PubMed Central PMCID: PMC1489311. 16820507 10.1128/AEM.03006-05 PMC1489311

[27] Wang Q, Garrity GM, Tiedje JM, Cole JR. Naive Bayesian classifier for rapid assignment of rRNA sequences into the new bacterial taxonomy. Applied and environmental microbiology. 2007;73(16):5261–7. doi: 10.1128/AEM.00062-07 ; PubMed Central PMCID: PMC1950982. 17586664 10.1128/AEM.00062-07 PMC1950982

[28] Chiu CJ, McArdle AH, Brown R, Scott HJ, Gurd FN. Intestinal mucosal lesion in low-flow states. I. A morphological, hemodynamic, and metabolic reappraisal. Archives of surgery. 1970;101(4):478–83. . 5457245 10.1001/archsurg.1970.01340280030009

[29] White JR, Nagarajan N, Pop M. Statistical methods for detecting differentially abundant features in clinical metagenomic samples. PLoS Comput Biol. 2009;5(4):e1000352 doi: 10.1371/journal.pcbi.1000352 ; PubMed Central PMCID: PMCPMC2661018. 19360128 10.1371/journal.pcbi.1000352 PMC2661018

[30] Allen-Blevins CR, You X, Hinde K, Sela DA. Handling stress may confound murine gut microbiota studies. PeerJ. 2017;5:e2876 doi: 10.7717/peerj.2876 ; PubMed Central PMCID: PMCPMC5234434. 28097073 10.7717/peerj.2876 PMC5234434

[31] Takala J. Determinants of splanchnic blood flow. Br J Anaesth. 1996;77(1):50–8. . 8703630 10.1093/bja/77.1.50

[32] Hsiao JK, Huang CY, Lu YZ, Yang CY, Yu LC. Magnetic resonance imaging detects intestinal barrier dysfunction in a rat model of acute mesenteric ischemia/reperfusion injury. Invest Radiol. 2009;44(6):329–35. doi: 10.1097/RLI.0b013e3181a16762 . 19363446 10.1097/RLI.0b013e3181a16762

[33] Nagatomo Y, Tang WH. Intersections Between Microbiome and Heart Failure: Revisiting the Gut Hypothesis. J Card Fail. 2015;21(12):973–80. doi: 10.1016/j.cardfail.2015.09.017 ; PubMed Central PMCID: PMCPMC4666782. 26435097 10.1016/j.cardfail.2015.09.017 PMC4666782

[34] Seropian IM, Sonnino C, Van Tassell BW, Biasucci LM, Abbate A. Inflammatory markers in ST-elevation acute myocardial infarction. European heart journal Acute cardiovascular care. 2015 doi: 10.1177/2048872615568965 . 25681486 10.1177/2048872615568965

[35] Cani PD, Amar J, Iglesias MA, Poggi M, Knauf C, Bastelica D, et al Metabolic endotoxemia initiates obesity and insulin resistance. Diabetes. 2007;56(7):1761–72. doi: 10.2337/db06-1491 . 17456850 10.2337/db06-1491

[36] Clemente-Postigo M, Queipo-Ortuno MI, Murri M, Boto-Ordonez M, Perez-Martinez P, Andres-Lacueva C, et al Endotoxin increase after fat overload is related to postprandial hypertriglyceridemia in morbidly obese patients. Journal of lipid research. 2012;53(5):973–8. doi: 10.1194/jlr.P020909 ; PubMed Central PMCID: PMC3329396. 22394503 10.1194/jlr.P020909 PMC3329396

[37] Yu XL, Chan Y, Zhuang LF, Lai HC, Lang NP, Lacap-Bugler DC, et al Distributions of Synergistetes in clinically-healthy and diseased periodontal and peri-implant niches. Microb Pathog. 2016;94:90–103. doi: 10.1016/j.micpath.2015.11.029 . 26686411 10.1016/j.micpath.2015.11.029

[38] Samuel BS, Gordon JI. A humanized gnotobiotic mouse model of host-archaeal-bacterial mutualism. Proceedings of the National Academy of Sciences of the United States of America. 2006;103(26):10011–6. doi: 10.1073/pnas.0602187103 ; PubMed Central PMCID: PMC1479766. 16782812 10.1073/pnas.0602187103 PMC1479766

[39] Cheng P, Zeng W, Li L, Huo D, Zeng L, Tan J, et al PLGA-PNIPAM Microspheres Loaded with the Gastrointestinal Nutrient NaB Ameliorate Cardiac Dysfunction by Activating Sirt3 in Acute Myocardial Infarction. Adv Sci (Weinh). 2016;3(12):1600254 doi: 10.1002/advs.201600254 ; PubMed Central PMCID: PMCPMC5157182. 27981013 10.1002/advs.201600254 PMC5157182

[40] Nakamura S, Islam MS. Motility of Spirochetes. Methods Mol Biol. 2017;1593:243–51. doi: 10.1007/978-1-4939-6927-2_19 . 28389959 10.1007/978-1-4939-6927-2_19

[41] Marcinkiewicz AL, Kraiczy P, Lin YP. There Is a Method to the Madness: Strategies to Study Host Complement Evasion by Lyme Disease and Relapsing Fever Spirochetes. Frontiers in microbiology. 2017;8:328 doi: 10.3389/fmicb.2017.00328 ; PubMed Central PMCID: PMCPMC5332432. 28303129 10.3389/fmicb.2017.00328 PMC5332432

[42] Alauzet C, Marchandin H, Courtin P, Mory F, Lemee L, Pons JL, et al Multilocus analysis reveals diversity in the genus Tissierella: description of Tissierella carlieri sp. nov. in the new class Tissierellia classis nov. Syst Appl Microbiol. 2014;37(1):23–34. doi: 10.1016/j.syapm.2013.09.007 . 24268443 10.1016/j.syapm.2013.09.007

