# Supplementary figures and images for: The changes of gut microbiota after acute myocardial infarction in rats
Source: PLoS One. 2017 Jul 7;12(7):e0180717. doi: 10.1371/journal.pone.0180717 (PMC5501596; doi:10.1371/journal.pone.0180717)

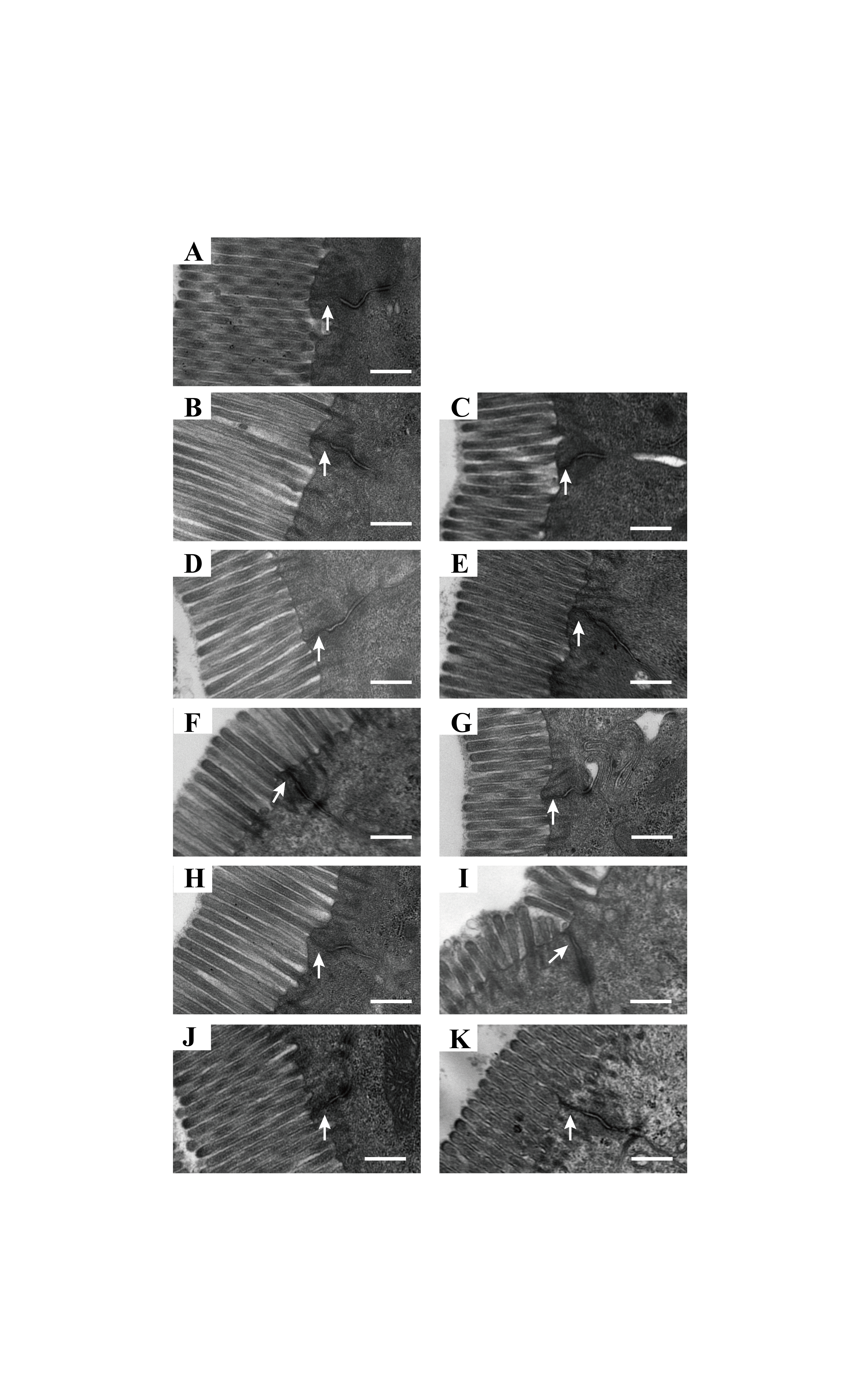

Supplement: S1 Fig — Transmission electron microscopy of ultrastructures of ileum (bar = 2.5 μm) showed that microvilli became short, sparse, irregularly arranged at 7 d post-AMI (white arrow shows the tight junction). A. Control group; B, D, F, H, J represents SHAM group at 12 h, 1 d, 3 d, 7 d, 14 d respectively; C, E, G, I, K represents AMI group at 12 h, 1 d, 3 d, 7 d, 14 d respectively. (TIF) [file pone.0180717.s001.tif]

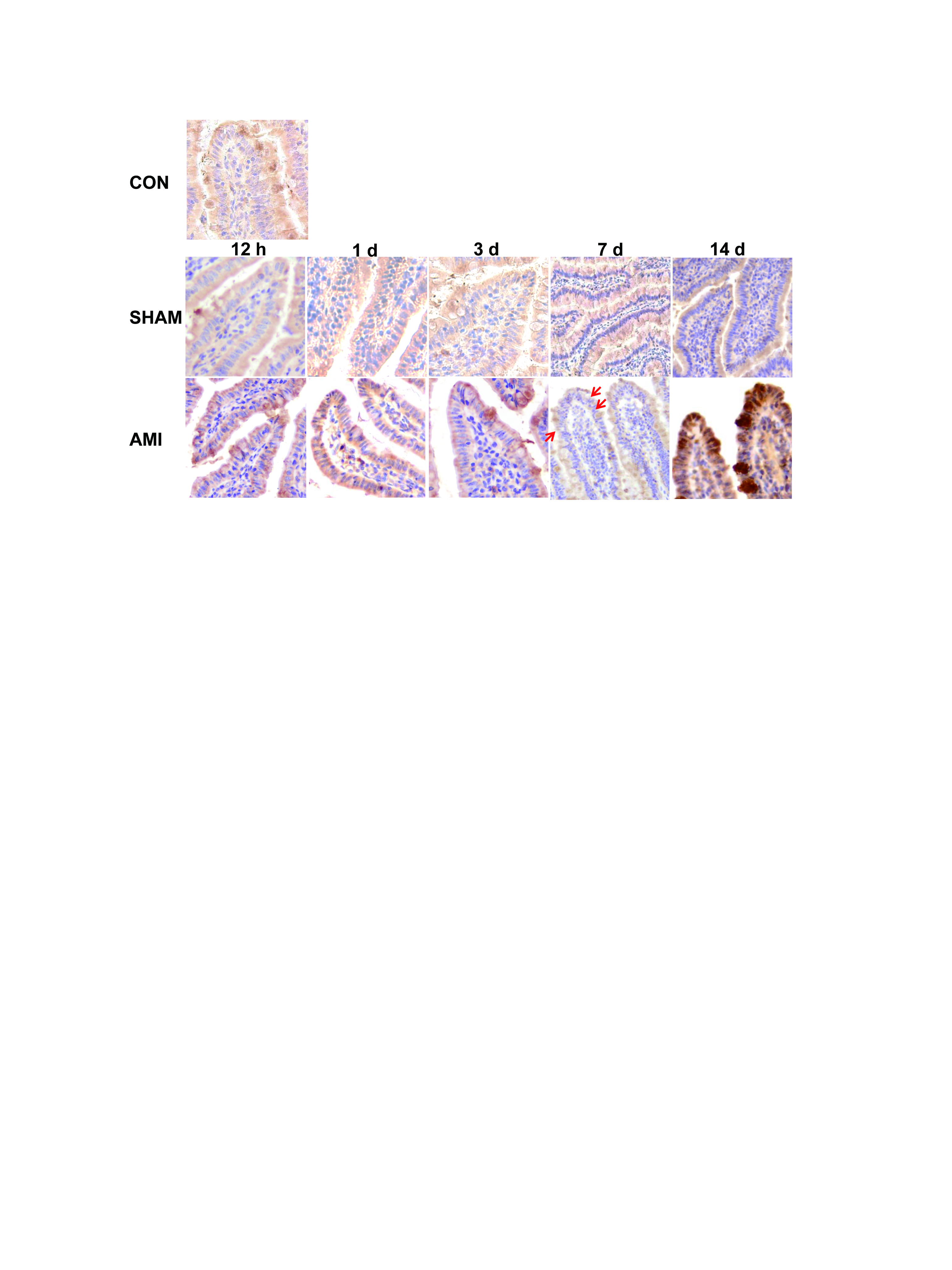

Supplement: S2 Fig — Immunohistochemical stain of tight junction protein occludin showed reduced expression of occludin (red arrow) in AMI group at 7 d post AMI. (TIF) [file pone.0180717.s002.tif]
